# MRE11 orchestrates porcine oocyte meiotic progression by modulating the spindle assembly checkpoint

**DOI:** 10.3389/fcell.2025.1635110

**Published:** 2025-08-08

**Authors:** Dandan Zhang, Zaishan Yang, Yongteng Zhang, Fugui Fang, Hongguo Cao, Yunsheng Li, Zubing Cao, Yanfeng Xue, Mianqun Zhang

**Affiliations:** ^1^ Department of Reproductive Medicine, General Hospital of Wanbei Coal Group, Suzhou, China; ^2^ Key Laboratory of Local Livestock and Poultry Genetical Resource Conservation and Breeding of Anhui Province, College of Animal Science and Technology, Anhui Agricultural University, Hefei, China

**Keywords:** MRE11, oocytes development, SAC, spindle, actin

## Abstract

**Introduction:**

Mre11 is a multisubunit nuclease involved in DNA repair, and its dysfunction often causes DNA damage sensitivity, genomic instability, telomere shortening, and aberrant meiosis. However, the specific roles of Mre11 in porcine oocyte meiosis remain unclear.

**Methods:**

In this study, porcine oocytes were treated with the Mre11-specific inhibitor mirin to investigate the function of Mre11 during meiotic maturation. Meiotic progression, spindle and chromosome structure, spindle migration, cytoplasmic actin polymerization, and DNA damage levels were assessed using immunofluorescence and relevant molecular markers including BubR1 and γH2A.X.

**Results:**

Inhibition of Mre11 activity led to failure of first polar body extrusion, with sustained BubR1 presence at kinetochores, indicating activation of the spindle assembly checkpoint (SAC). Mre11-inhibited oocytes showed disrupted spindle and chromosome organization due to decreased microtubule stability. Additionally, spindle migration to the oocyte cortex was impaired, correlating with reduced cytoplasmic actin polymerization. Elevated DNA damage levels were observed in treated oocytes as evidenced by increased γH2A.X staining.

**Discussion:**

These findings demonstrate that Mre11 is essential for porcine oocyte meiotic progression by maintaining normal spindle assembly, actin cytoskeleton dynamics, and SAC activity. DNA damage accumulation following Mre11 inhibition likely contributes to meiotic failure, highlighting its critical role in ensuring oocyte quality.

## Introduction

Oocyte meiosis involves a single round of DNA replication followed by two sequential chromosome segregation events: meiosis I and meiosis II. (Morgan et al., 2023; Petronczki et al., 2003; Mullen et al., 2019; Mogessie et al., 2018). During this process, the spindle assembly checkpoint (SAC) proteins play a crucial role (Dou et al., 2019). The spindle assembly checkpoint (SAC) ensures accurate chromosome segregation by preventing the metaphase-anaphase transition until all chromosomes are correctly attached to the spindle microtubules. Impaired SAC activity accelerates the meiotic process in oocytes, leading to the formation of aneuploid eggs (McAinsh and Kops, 2023; Duro and Nilsson, 2021; Shimoi et al., 2019; Musacchio and Salmon, 2007; Lara-Gonzalez et al., 2012).

DNA double-strand breaks (DSBs) are among the most lethal types of DNA damage, threatening cell viability if unrepaired. DSBs play a pivotal role in various biological processes, including chromosomal recombination during meiosis in germ cells (Bauer et al., 2015; Baudat et al., 2013). One of the key complexes responsible for recognizing, repairing, and signaling DSBs is the MRN/X complex, composed of the Mre11, Rad50, and Nbs1/Xrs2 proteins. Mre11 is the nuclease component of the MRN/X complex, and it is highly conserved across all organisms (Girard et al., 2018; Vertemara and Tisi, 2023; Paull, 2018; Rotheneder et al., 2023). Its critical role in DNA double-strand break repair and recombination was first established through genetic studies in *Saccharomyces cerevisiae* (Lettier et al., 2006; Johzuka and Ogawa, 1995). Early studies in budding yeast revealed that Mre11 and its nuclease activity are indispensable for processing Spo11-covalently bound breaks during meiosis, which are crucial for initiating homologous recombination (Borner et al., 2023; Mimitou and Symington, 2009; Hodgson et al., 2011). Mre11 is capable of removing various nucleic acid and protein blocks from DNA ends. Additionally, under certain pathological conditions, the nuclease activity of Mre11 can have deleterious effects on DNA replication intermediates (Paull and Gellert, 1998).

It is now understood that the Mre11 complex initiates the processing of DNA double-strand breaks (DSBs) in a variety of cellular contexts beyond meiosis (Petroni et al., 2018; Reginato and Cejka, 2020). Previous studies have shown that mice expressing a nuclease-deficient form of Mre11 are unable to survive early embryogenesis, as the loss of Mre11 nuclease activity is lethal in both mice and cultured vertebrate cells (Buis et al., 2008; Xiao and Weaver, 1997; Spehalski et al., 2017). Mammalian cells lacking Mre11 nuclease activity rapidly exhibit spontaneous chromosomal abnormalities, including chromatid breaks, translocations, dicentric chromosomes, and radial structures (Stracker et al., 2004; D'Amours and Jackson, 2002). Furthermore, research has demonstrated that human TK6 cells and chicken DT40 cells deficient in Mre11 nuclease activity accumulate covalent Top2 adducts, a type of damage typically observed only in cells treated with Top2 poisons (Hoa et al., 2016; Shimizu et al., 2020). The overexpression of Tdp2, an enzyme specifically involved in the removal of Top2-DNA damage, can delay and partially suppress the lethality of Mre11 nuclease-deficient cells, indicating that these specific protein-DNA adducts contribute to the observed phenotype. Interestingly, studies have also found that Mre11 nuclease activity is crucial for the repair of DSBs during the G1 phase of the cell cycle (Schellenberg et al., 2016; Sasanuma et al., 2020; Takeda et al., 2016; Šamanić et al., 2016; Dasika et al., 1999; Deshpande et al., 2016; Wojtaszek and Williams, 2024). Despite its well-characterized role in DNA repair and genome stability, the specific function of Mre11 during mammalian oocyte meiosis remains poorly understood.

To address this gap, we utilized porcine oocytes-an established model with high physiological relevance to humans-to explore potential roles of Mre11 in meiosis beyond its canonical function in DNA repair. We investigated the potential roles of Mre11 during oocyte meiosis beyond its known function in DNA damage repair. Specifically, we examined the localization and expression patterns of Mre11 at various developmental stages of meiosis. Additionally, we employed the Mre11-specific inhibitor mirin to assess the impact of Mre11 activity on polar body extrusion, spindle assembly checkpoint function, microtubule stability, and DNA damage levels in porcine oocytes.

## Materials and methods

### Antibodies

Rabbit polyclonal anti-MRE11 antibody was purchased from Proteintech Group (Rosemont, IL, United States; Cat# 16370-1-AP); rabbit monoclonal anti-BUBR1 antibody was purchased from Abcam (Cambridge, United Kingdom, United States; Cat# ab133699); Mouse anti-α-tubulin-FITC antibody (Cat# F2168), mouse monoclonal anti-acetylation-α-tubulin antibody (T7451), peanut agglutinin (PNA)-FITC (L7381) and DAPI (D9542) were purchased from Sigma (Aldrich, St Louis, MO, United States); Rabbit polyclonal anti-γ-H2AX antibody (Cat# ab26350) was purchased from Abcam (Cambridge, United Kingdom); TRITC Phalloidin TRITC antibody (Cat# MX4405) was purchased from Maokang Biotechnology (Shanghai, China); Furthermore, goat anti-mouse IgG Alexa Fluor 488 antibody (Cat# A11029), donkey anti-Sheep IgG Alexa Fluor 594 antibody (Cat# A11016) were purchased from Thermo Fisher (Waltham, MA, United States). human anti-centromere antibody was purchased from Antibodies Incorporated (Davis, CA, United States; Cat# CA95617); rabbit monoclonal anti-GAPDH antibody was purchased from Cell Signaling Technology (Danvers, MA, United States; Cat# 2118).

### Collection and culture of porcine oocytes

Ovaries were collected from pigs at a local abattoir and transported to the laboratory within 2 h post-slaughter in sterile 0.9% saline supplemented with streptomycin sulfate and penicillin G. Cumulus-oocyte complexes (COCs) were aspirated from follicles using a disposable syringe. Only COCs with intact and compact cumulus cell layers were selected for *in vitro* maturation (IVM). The maturation medium consisted of TCM-199 (ThermoFisher Scientific, Waltham, MA, United States; Cat# 11150059), supplemented with 10% porcine follicular fluid, 5 μg/mL insulin, 10 ng/mL epidermal growth factor (EGF), 0.6 mM cysteine, 0.2 mM pyruvate, 25 μg/mL kanamycin, and 10 IU/mL of both equine chorionic gonadotropin (eCG) and human chorionic gonadotropin (hCG). Groups of 20 germinal vesicle (GV) stage COCs were cultured in 100 μL droplets of maturation medium under mineral oil at 38.5°C in a humidified atmosphere 5% CO_2_ for 26–28 h to reach metaphase I, and for 42–44 h to reach metaphase II.

### Mirin treatment

The mirin compound (Borde and Cobb, 2009) was dissolved in dimethyl sulfoxide (DMSO) and subsequently diluted into the culture medium to achieve final working concentrations of 20, 40, 60, or 100 μmol/L. Porcine oocytes at the germinal vesicle (GV), germinal vesicle breakdown (GVBD), metaphase I (MI), and metaphase II (MII) stages were collected after incubation in maturation medium containing mirin for 0, 20, 28, or 44 h, respectively.

### Immunofluorescence and measurement of fluorescence intensity

Denuded oocytes (DOs) were fixed in 4% paraformaldehyde in PBS for 30 min, permeabilized with 1% Triton X-100 in PBS for 1 h, and blocked with 1% bovine serum albumin (BSA) in PBS at room temperature for 1 h. The oocytes were then incubated overnight at 4°C with primary antibodies against MRE11 (1:100), BUBR1 (1:100), centromeres (1:200), and α-tubulin-FITC (1:200). After washing with PBST (PBS with 0.1% Tween-20), the oocytes were incubated with the appropriate secondary antibodies for 1 h at room temperature, followed by counterstaining with 10 μg/mL Hoechst 33342 or propidium iodide (PI) for 10 min. The oocytes were then mounted on slides and visualized using a confocal microscope (LSM 700 META, Zeiss, Germany).

### Nocodazole treatment of oocytes

For nocodazole treatment, a stock solution of nocodazole (10 mg/mL in DMSO) was diluted in M16 medium (Sigma-Aldrich) to a final concentration of 20 μg/mL. Oocytes were incubated in the nocodazole-supplemented M16 medium for 10 min, followed by an additional 9 h of incubation at 38.5°C in 5% CO_2_. Afterward, the oocytes were collected for immunofluorescence microscopy.

## Quantification of fluorescence signals

Oocytes subjected to standardized immunofluorescence staining were imaged on the same day using a Zeiss confocal laser scanning microscope (e.g., LSM 880, Zeiss, Germany). To minimize batch effects, all imaging parameters—including laser intensity, scan speed, pinhole size, gain, and offset—were kept constant. Images were exported in TIFF format and analyzed using ImageJ software (v1.53o, NIH, United States). A consistent threshold was applied to remove background signals. Regions of interest (ROIs) were selected using the ROI Manager to delineate the cytoplasmic or nuclear area. Total fluorescence intensity within each ROI was measured and normalized to its area to obtain the mean gray value (mean intensity/μm^2^). At least 20 oocytes or equivalent tissue regions were analyzed per group, and the average value was used for subsequent comparisons.

### Statistical analysis

All values, including percentages, were derived from at least three independent experiments and are presented as the mean ± SEM or mean ± SD, with the number of oocytes analyzed indicated in parentheses (n). Data were statistically analyzed using a a two-tailed paired t-test, performed with GraphPad Prism 5 software. A p-value of less than 0.05 (P < 0.05) was considered statistically significant.

## Results

### Localization and expression patterns of Mre11 in porcine oocytes

**FIGURE 1:**
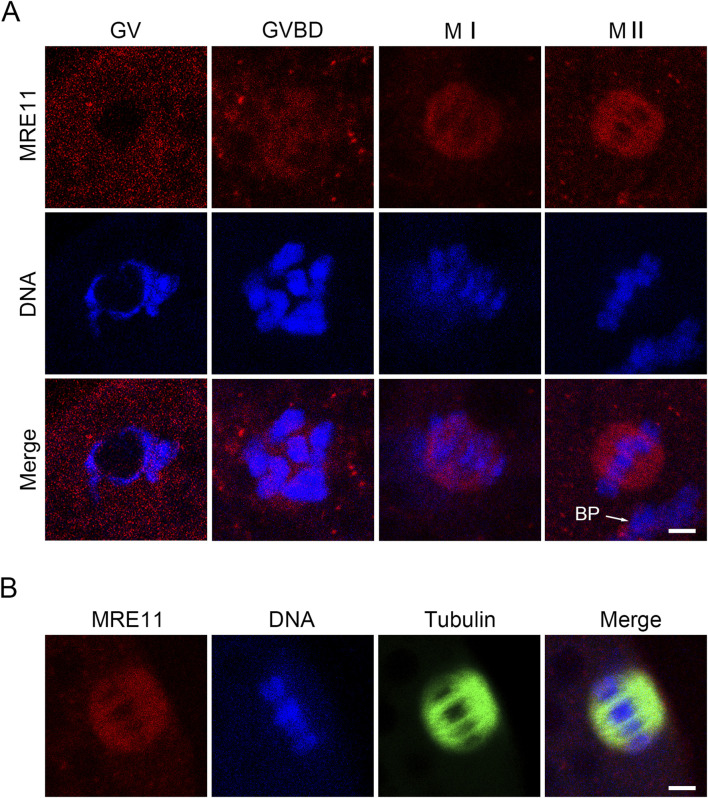
Subcellular localization and protein expression of Mre11 during porcine meiotic maturation. **(A)** Representative immunofluorescence images showing the localization of endogenous Mre11 in porcine oocytes at different developmental stages. Oocytes were immunostained with an anti-Mre11 antibody and counterstained with Hoechst. Scale bar, 2.5 μm. **(B)** Representative image showing the co-localization of Mre11 with kinetochores in porcine oocytes at the pro-metaphase I (Pro-M I) stage. Oocytes were immunostained with anti-Mre11 and CREST antibodies, and counterstained with Hoechst. Scale bar, 2.5 μm.

To investigate the role of Mre11 in porcine oocyte meiotic maturation, we examined its cellular localization and expression levels at distinct meiotic stages. Germinal vesicle (GV) stage oocytes were freshly isolated, and oocytes cultured for 20, 28, and 44 h -corresponding to germinal vesicle breakdown (GVBD), metaphase I (MI), and metaphase II (MII) stages-were subjected to immunofluorescence staining. Our results demonstrated that Mre11 consistently localizes to the spindle apparatus throughout oocyte maturation, suggesting its potential involvement in spindle assembly during meiosis (Figures 1A,B).

### Mre11 is essential for the meiotic progression of porcine oocytes

**FIGURE 2:**
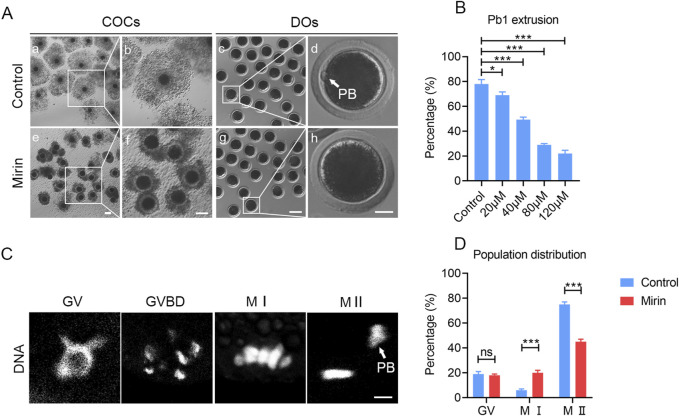
Effect of Mre11 inhibition on porcine oocyte meiotic progression. **(A)** Representative images of cumulus cell expansion and polar body extrusion in control and Mre11-inhibited oocytes cultured *in vitro* for 44 h. Oocytes were denuded after culture to observe polar body extrusion. COCs: cumulus-oocyte complexes; DOs: denuded oocytes. Scale bars, 500 μm (a, e); 200 μm (b, f); 250 μm (c, g); 30 μm (d, h). **(B)** The rate of polar body extrusion was recorded in control and Mre11-inhibited groups treated with different concentrations (20 μM, 40 μM, 60 μM, and 100 μM) after 44 h of culture. **(C)** Representative images showing chromosome morphology at different developmental stages of oocyte maturation. DNA was counterstained with propidium iodide (PI). Scale bar, 5 μm. **(D)** The percentage of oocytes at different developmental stages was quantified in control and Mre11-inhibited groups. Data in panels **(B)** and **(D)** are presented as mean percentages (mean ± SEM) from at least three independent experiments. *P < 0.05, **P < 0.01, ***P < 0.001.

To evaluate the effect of Mre11 inhibition on the meiotic progression of porcine oocytes, we employed the MRE11-specific inhibitor mirin to block MRE11 activity and subsequently assessed the frequency of polar body extrusion (PBE), a critical developmental event indicating the completion of meiosis I. As shown in Figures 2A,B, after 42–44 h of *in vitro* culture with varying doses of mirin (20, 40, 60, and 100 μmol/L), we observed that the incidence of PBE in Mre11-depleted oocytes was significantly higher than in the control group (control: 78% ± 2.08%, n = 121; 20 μmol/L: 69% ± 1.528%, n = 109, P < 0.05; 40 μmol/L: 49.33% ± 1.202%, n = 114, P < 0.05; 60 μmol/L: 29% ± 0.57%, n = 100, P < 0.05; 100 μmol/L: 22% ± 1.52%,n = 89, P < 0.05; Figure 2B). Based on these results, 40 μmol/L mirin was chosen for subsequent experiments due to its marked inhibitory effect. Furthermore, as shown in Figures 2C,D, karyotype analysis of the oocytes revealed that most MRE11-inhibited oocytes that failed to extrude a polar body were arrested at metaphase I (MI) (control:6% ± 0.5%, n = 33vs Mirin: 20% ± 2%, n = 40, P < 0.5; Figure 2D).

### Inhibition of Mre11 leads to the activation of spindle checkpoint in porcine oocytes

**FIGURE 3:**
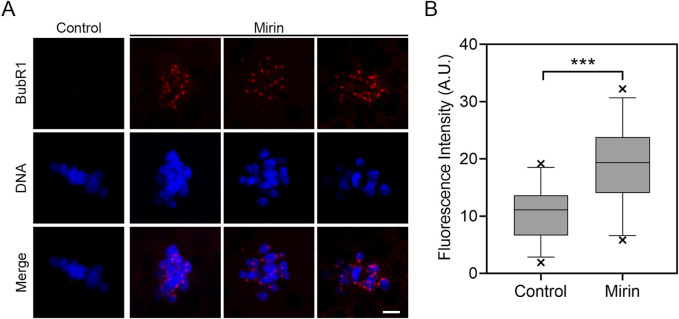
Effect of Mre11 inhibition on BubR1 localization in porcine oocytes. **(A)** Representative images showing the localization of BubR1 at the metaphase I stage in control and Mre11-inhibited oocytes. Scale bar, 5 μm. **(B)** Quantitative analysis of BubR1 fluorescence intensity in control and Mre11-inhibited oocytes. Data are presented as the mean percentage (mean ± SEM) from at least three independent experiments. **P < 0.001.

To determine whether the MI arrest during oocyte development is caused by the activation of the spindle assembly checkpoint (SAC), we performed immunostaining for BubR1, a key component of the SAC complex, in Mre11-inhibited oocytes. Immunofluorescence analysis revealed that in control oocytes, BubR1 was absent from the chromosomes at the MI stage, permitting progression into anaphase. However, in Mre11-inhibited oocytes, BubR1 remained localized at the kinetochores with strong signals (Figure 3A). Consistent with this observation, quantification of fluorescence intensity demonstrated that BubR1 levels were significantly higher in Mre11-inhibited oocytes compared to controls (control:10.43 ± 0.8,n = 30 vs Mirin: 18.92 ± 1.32, n = 27, P < 0.05; Figure 3B), suggesting that the MI arrest in these oocytes is a consequence of SAC activation.

To further investigate the role of Mre11 in SAC activation, we also examined the resistance of oocytes to the microtubule depolymerizing agent nocodazole. In control oocytes, although the spindle structure collapsed after 5 min of nocodazole treatment, residual microtubules were still detectable. In contrast, microtubules in Mre11-depleted oocytes were completely depolymerized following the same treatment, indicating reduced microtubule stability, which likely contributes to SAC activation.

### Mre11 is essential for the spindle/chromosome structure in porcine oocytes

**FIGURE 4:**
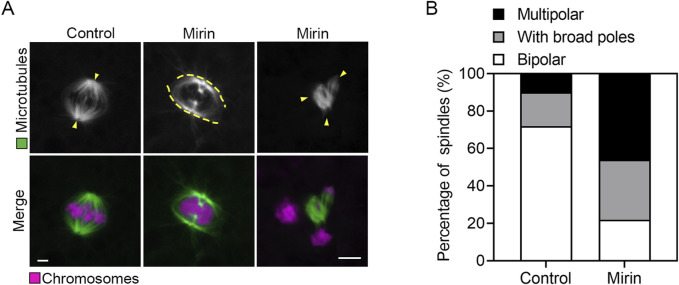
Effect of Mre11 inhibition on spindle assembly and chromosome alignment in porcine oocytes. **(A)** Representative images depicting spindle morphologies and chromosome alignment in control and Mre11-inhibited oocytes. Metaphase I (M I) oocytes were immunostained with anti-α-tubulin-FITC antibody to visualize spindles and counterstained with propidium iodide (PI) to visualize chromosomes. Scale bar, 5 μm. **(B)** The frequency of aberrant spindles was recorded in control and Mre11-inhibited oocytes. Data in panel **(B)** are presented as mean percentages (mean ± SEM) from at least three independent experiments.

Given the observed spindle assembly defects typically resulting from impaired SAC activity, we next examined whether similar defects occur in Mre11-depleted porcine oocytes. To investigate this, we performed immunostaining of MI-stage oocytes from both control and Mre11-depleted groups using an anti-α-tubulin antibody to visualize spindle structures, and counterstained with PI to display chromosomal alignment. As shown in Figure 4A, control oocytes exhibited well-organized spindle apparatuses with chromosomes properly aligned at the equatorial plate. In contrast, Mre11-depleted oocytes displayed a significantly increased frequency of spindle disorganization and chromosome misalignment, characterized by a variety of abnormal spindle morphologies. The predominant abnormalities observed included broad-polar spindles and multipolar spindles (control:18% ± 1.5%, n = 83 vs. Mirin: 32% ± 0.8%, n = 75, P < 0.05, with broad poles; control:10% ± 2%, n = 88 vs. Mirin: 46% ± 3.5%, n = 90, p < 0.05, multipolar; Figure 4B).

### Inhibition of Mre11 weakens the microtubule stability in porcine oocytes

**FIGURE 5:**
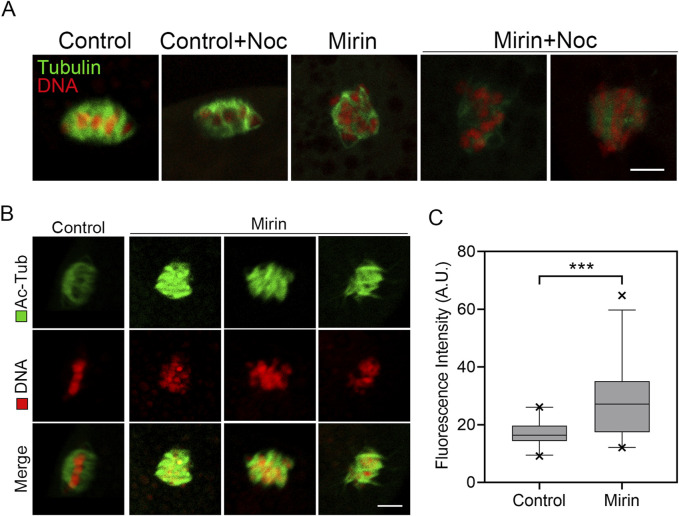
Effect of Mre11 inhibition on the acetylation level of α-tubulin in porcine oocytes. **(A)** Representative images of microtubule fibers before and after nocodazole treatment in control and Mre11-inhibited oocytes. Oocytes were immunostained with an anti-α-tubulin-FITC antibody to visualize microtubules and counterstained with propidium iodide (PI) to visualize chromosomes. Scale bar, 10 μm. **(B)** Representative images showing the acetylation of α-tubulin in control and Mre11-inhibited oocytes. Oocytes were immunostained with an anti-acetyl-α-tubulin (Lys-40) antibody to assess the acetylation level of α-tubulin. Scale bar, 5 μm. **(C)** The fluorescence intensity of acetylated α-tubulin was quantified in control and Mre11-inhibited oocytes. Data in panel **(C)** are presented as mean percentages (mean ± SEM) from at least three independent experiments. ***P < 0.001.

Since spindle assembly is regulated by microtubule dynamics, we investigated whether the abnormal spindle organization observed in MRE11-inhibited oocytes is due to defects in microtubule stability. To test this hypothesis, we used the microtubule depolymerizing agent nocodazole to disrupt microtubules. As shown in Figure 5A, in control oocytes, microtubule fibers remained present after 5 min of nocodazole treatment, although spindle morphology was disrupted. However, in MRE11-inhibited oocytes, microtubule fibers completely disappeared following the same treatment, indicating reduced microtubule stability. This observation was further validated by assessing the acetylation level of α-tubulin (Figure 5B), a known marker of microtubule stability that has been previously reported in oocytes. Through immunofluorescence analysis and quantification of fluorescence intensity, a significant reduction in acetylated α-tubulin levels was observed in MRE11-inhibited oocytes compared to controls. Overall (control:16.9 ± 0.8, n = 25 vs. Mirin: 27.74 ± 2.5, n = 25, p < 0.001; Figure 5C), these data suggest that MRE11 inhibition compromises microtubule stability, thereby disrupting normal spindle assembly during meiotic maturation in oocytes.

### Inhibition of Mre11 impairs actin dynamics in porcine oocytes

**FIGURE 6:**
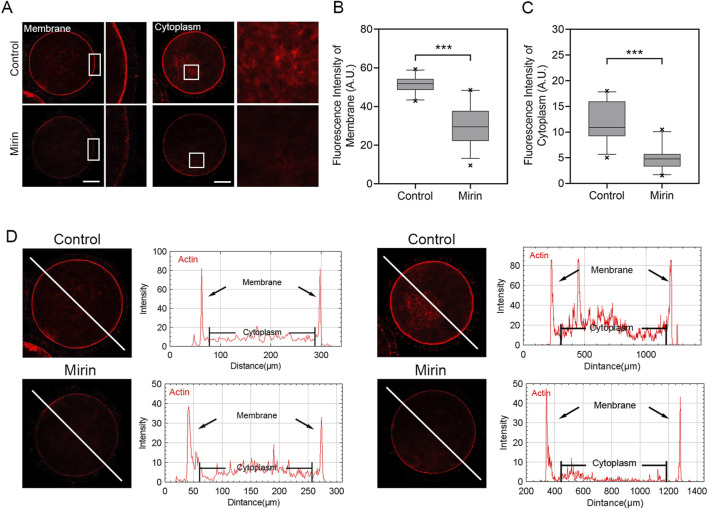
Effect of Mre11 inhibition on cytoplasmic actin assembly in porcine oocytes. **(A)** Representative images showing the distribution of actin filaments at the oocyte cortex and cytoplasm in the control and Mre11-inhibited groups. Red indicates F-actin. Scale bar, 20 μm. **(B)** Quantification of fluorescence intensity of F-actin at the cortex in the control and Mre11-inhibited groups. **(C)** Quantification of fluorescence intensity of F-actin in the cytoplasm in the control and Mre11-inhibited groups. **(D)** Quantitative analysis of F-actin fluorescence intensities at the cortex and in the cytoplasm. Data in panels **(B–D)** are presented as mean percentages (mean ± SEM) from at least three independent experiments. ***P < 0.001.

To investigate the cause of meiotic arrest induced by Mre11 inhibition, we further examined the distribution of actin, as the failure of actin-mediated spindle migration could lead to unsuccessful polar body extrusion in oocytes. As shown in Figure 6A, the actin signals in control porcine oocytes were enriched around the spindle area and evenly distributed across the plasma membrane. In contrast, the levels of F-actin in the plasma membrane and cytoplasm were significantly reduced in Mre11-inhibited porcine oocytes. Furthermore, quantitative analysis indicated that Mre11 inhibition significantly decreased the fluorescence intensity (control: 51.38 ± 0.8, n = 26 vs Mirin: 30.46.±1.9, n = 28, P < 0.001, membrane; control: 12 ± 0.7, n = 27 vs Mirin: 4.8 ± 0.4, n = 26,P < 0.001; Figures 6B,C,D).

### Inhibition of Mre11 impairs spindle migration in porcine oocytes

**FIGURE 7:**
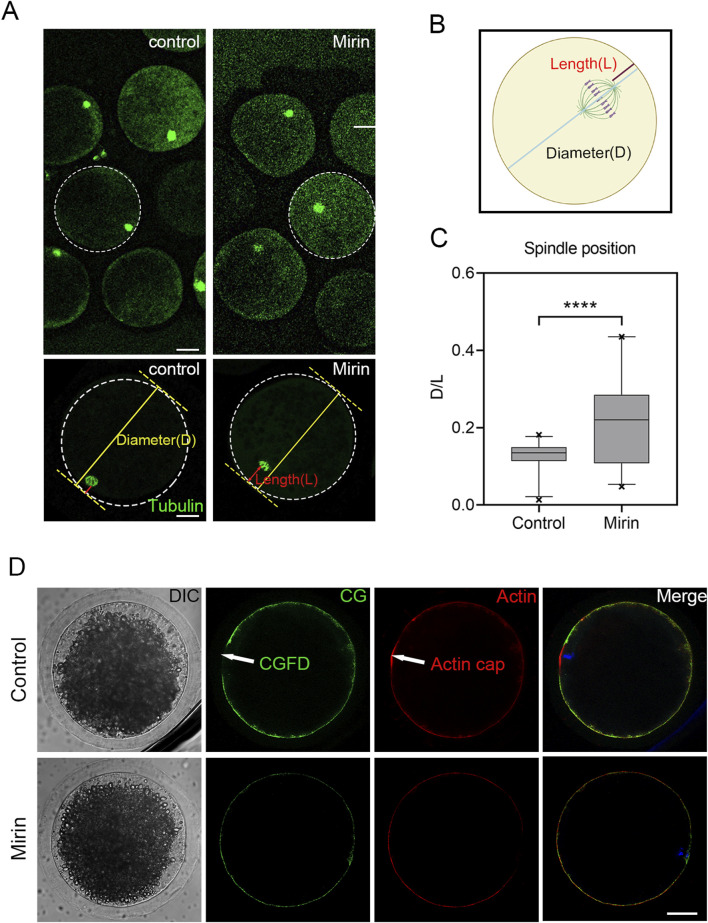
Effect of Mre11 inhibition on meiotic spindle positioning and the formation of actin caps in porcine oocytes. **(A)** Representative images showing spindle positioning in control and Mre11-inhibited oocytes. **(B)** To quantify spindle position after Mre11 inhibition, D is defined as the distance from the proximal end of the spindle pole to the cortex, while L is defined as the distance from the distal end of the spindle pole to the cortex. **(C)** The D/L ratio in the Mre11-inhibited group was significantly higher than that in the control group. **(D)** Formation of actin caps and CGFDs. After Mre11 inhibition, the actin cap over a chromosome was disrupted, and Mre11 inhibition also affected CGFD formation. Red indicates actin; green indicates CGs; blue indicates chromatin. Scale bar, 20 μm.

The meiotic arrest and failure of polar body extrusion may also be attributed to spindle migration defects caused by actin assembly dysregulation triggered by Mre11 inhibition. To investigate this, we assessed spindle positioning in porcine oocytes treated with miRIN after 28 h of culture, which is the time point when most oocyte spindles migrate to the cortex. As shown in Figure 7, most spindles in the control group migrated to the cortex, whereas in the miRIN-treated group, spindles remained at the center of the oocytes. Additionally, statistical analysis revealed that the spindle migration rate in the treatment group was significantly higher than that in the control group (control: 0.125 ± 0.007, n = 27 vs. Mirin:0.216 ± 0.022, n = 29, P < 0.001; Figure 7C). These results suggest that Mre11 may influence spindle migration in porcine oocytes, thereby affecting polar body formation and extrusion.

### Inhibition of Mre11 elevates the level of DNA damage in porcine oocytes

**FIGURE 8:**
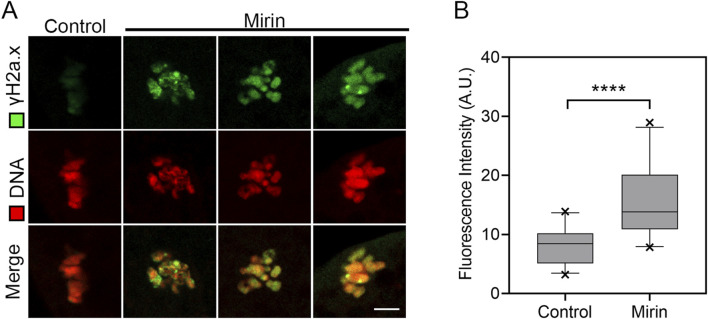
Effect of Mre11 inhibition on DNA damage levels in porcine oocytes. **(A)** Representative images showing DNA damage in control and Mre11-inhibited oocytes. Oocytes were immunostained with an anti-γH2A.X antibody and counterstained with Hoechst. Scale bar, 5 μm. **(B)** Quantification of γH2A.X fluorescence intensity in control and Mre11-inhibited oocytes. Data are presented as mean percentages (mean ± SEM) from at least three independent experiments. ***P < 0.001.

Studies have shown that Mre11 is localized at sites of DNA double-strand breaks, where it initiates and regulates the repair process of these breaks (Petroni et al., 2018; Yu et al., 2012; Borde and Cobb, 2009). Based on this function, we investigated whether inhibiting Mre11 would affect the level of DNA damage in oocytes. To test this, we performed immunofluorescence analysis using γH2A.X antibodies. The results indicated a significant accumulation of γH2A.X foci on chromosomes in Mre11-inhibited oocytes compared to the control group (Figure 8A), suggesting an increased level of DNA damage (control: 8.2 ± 0.6, n = 25 vs. Mirin: 15.5 ± 1.2, n = 26, P < 0.001; Figure 8B).

## Discussion

Given that the Mre11/Rad50 complex plays a critical role in maintaining genomic stability, Mre11, as the nuclease component of the MRN/X complex, has been a focal point of in-depth research (Zhang et al., 2022). Early studies in budding yeast have demonstrated that Mre11 and its nuclease activity are indispensable for processing covalent Spo11-linked DNA breaks during meiosis, which initiate homologous recombination (Borde, 2007; Usui et al., 1998). However, the specific role of Mre11 during meiosis in mammalian oocytes remains incompletely understood. Therefore, we utilized porcine oocytes as a model to explore the role of Mre11 in meiosis.

The localization pattern of Mre11 in porcine oocytes suggests that its function during oocyte meiosis may be related to spindle assembly and chromosome alignment. To test this hypothesis, we employed a Mre11-specific inhibitor to suppress Mre11 activity in porcine oocytes. Our results indicate that inhibition of Mre11 significantly reduces the frequency of polar body extrusion (PBE), thereby impairing the progression of oocyte meiosis, which suggests that Mre11 is essential for the normal maturation of porcine oocytes. Since Mre11 activity in cumulus cells was also blocked, the failure of oocyte maturation could be partially attributed to impaired cumulus cell expansion, although further studies are needed to clarify this effect. Further analysis of the arrested oocytes using DNA staining revealed that most Mre11-inhibited oocytes experienced meiotic failure at the metaphase I (MI) stage, implying that the spindle assembly checkpoint (SAC) may be activated to prevent the onset of anaphase. This hypothesis was confirmed by observing the persistent presence of BubR1, a core component of the SAC, at the kinetochores of Mre11-inhibited MI oocytes. Our results showed that 40 μmol/L mirin significantly increased BubR1 accumulation at kinetochores, suggesting SAC activation upon Mre11 inhibition. Although other concentrations were not tested, the evident SAC response at this dose supports its sufficiency for functional inhibition of Mre11. This provides further evidence for the critical role of Mre11 in meiotic spindle checkpoint regulation.

To investigate the cause of SAC activation during the MI stage, we examined the spindle and chromosome structures, which are known to be crucial for SAC regulation in oocytes (Polanski, 2013). As expected, we observed that inhibition of Mre11 significantly increased abnormalities in spindle morphology and chromosome alignment in porcine oocytes, suggesting that impaired spindle assembly may induce SAC activation during the MI stage. Such activation likely blocks the transition from metaphase to anaphase and consequently inhibits polar body extrusion.

In addition, the abnormal spindle organization prompted us to further assess microtubule dynamics. Both nocodazole treatment and levels of acetylated α-tubulin confirmed that microtubule stability was compromised in Mre11-inhibited oocytes, suggesting that defective microtubule dynamics may disrupt spindle assembly, thereby activating SAC and hindering the normal meiotic progression in porcine oocytes.

In addition to the effects of microtubule protein acetylation on spindle organization, we observed an interesting phenotype: Mre11 inhibition affects actin assembly and interferes with spindle migration. During meiosis, the migration of the spindle from the central region to the cortex is a critical asymmetric division process, essential for the oocyte to retain most of the maternal reserves for embryonic development. Spindle migration depends on the dynamics of cytoplasmic actin. Given the impact of Mre11 inhibition on cytoplasmic actin in this study, we analyzed spindle positioning and found that, that most spindles in Mre11-inhibited oocytes were arrested at the center, indicating defective migration toward the cortex. This may explain the occurrence of large polar bodies after Mre11 inhibition. We propose that Mre11 inhibition disrupts the actin network, thereby affecting spindle stability and positioning.

It has been reported that Mre11 facilitates the repair of DNA double-strand breaks (DSBs) in oocytes (Mayer et al., 2016). In the final set of results from our study, we found that the Mre11 inhibitor Mirin might block meiotic progression at the MI stage by affecting the DNA damage response in oocytes (Rahal et al., 2010). Thus, our findings support the hypothesis that inhibition of Mre11 leads to significant accumulation of DNA damage, which may represent another critical factor contributing to meiotic defects in porcine oocytes.

In summary, our findings provide evidence that Mre11 is essential for the meiotic progression of porcine oocytes by regulating spindle assembly and DNA damage repair. Inhibition of Mre11 impairs both of these processes, leading to the activation of the spindle assembly checkpoint (SAC) and meiotic arrest at the MI stage. Furthermore, our results not only improve understanding of potential causes of meiotic defects in human oocytes but also provide valuable insights, as porcine oocytes share greater developmental and physiological similarity to human oocytes than do mouse oocytes.

## Data Availability

The original contributions presented in the study are included in the article/Supplementary Material, further inquiries can be directed to the corresponding authors.
