# Supplementary figures and images for: MRE11 orchestrates porcine oocyte meiotic progression by modulating the spindle assembly checkpoint
Source: Front Cell Dev Biol. 2025 Aug 8;13:1635110. doi: 10.3389/fcell.2025.1635110 (PMC12370773; doi:10.3389/fcell.2025.1635110)

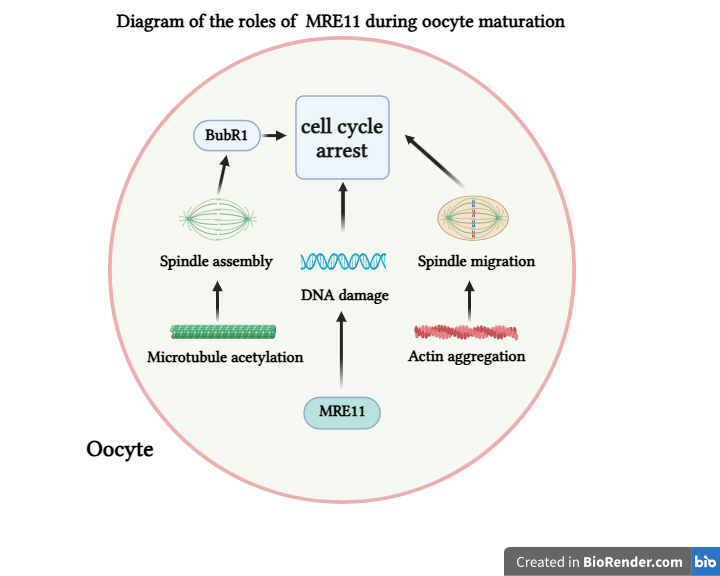

Supplement: Supplementary file 1 [file Image1.jpeg]
